# Robust and highly efficient hiPSC generation from patient non-mobilized peripheral blood-derived CD34^+^ cells using the auto-erasable Sendai virus vector

**DOI:** 10.1186/s13287-019-1273-2

**Published:** 2019-06-24

**Authors:** Takashi Okumura, Yumi Horie, Chen-Yi Lai, Huan-Ting Lin, Hirofumi Shoda, Bunki Natsumoto, Keishi Fujio, Eri Kumaki, Tsubasa Okano, Shintaro Ono, Kay Tanita, Tomohiro Morio, Hirokazu Kanegane, Hisanori Hasegawa, Fumitaka Mizoguchi, Kimito Kawahata, Hitoshi Kohsaka, Hiroshi Moritake, Hiroyuki Nunoi, Hironori Waki, Shin-ichi Tamaru, Takayoshi Sasako, Toshimasa Yamauchi, Takashi Kadowaki, Hiroyuki Tanaka, Sachiko Kitanaka, Ken Nishimura, Manami Ohtaka, Mahito Nakanishi, Makoto Otsu

**Affiliations:** 1 Division of Stem Cell Processing/Stem Cell Bank, Center for Stem Cell Biology and Regenerative Medicine, The Institute of Medical Science, The University of Tokyo, 4-6-1 Shirokanedai, Minato-ku, Tokyo, 108-8639 Japan; 2 Department of Allergy and Rheumatology, Graduation School of Medicine, The University of Tokyo, Tokyo, Japan; 3 Department of Pediatrics and Developmental Biology, Graduate School of Medical and Dental Sciences, Tokyo Medical and Dental University, Tokyo, Japan; 4 Department of Child Health and Development, Graduate School of Medical and Dental Sciences, Tokyo Medical and Dental University, Tokyo, Japan; 5 Department of Rheumatology, Graduate School of Medical and Dental Sciences, Tokyo Medical and Dental University, Tokyo, Japan; 6 Division of Rheumatology and Allergy, Department of Internal Medicine, St. Marianna University School of Medicine, Kawasaki, Kanagawa Japan; 7 Division of Pediatrics, Faculty of Medicine, University of Miyazaki, Miyazaki, Japan; 8 Department of Diabetes and Metabolic Diseases, Graduate School of Medicine, The University of Tokyo, Tokyo, Japan; 9 Department of Molecular Sciences on Diabetes, Graduate School of Medicine, The University of Tokyo, Tokyo, Japan; 10 Department of Prevention of Diabetes and Life-style Related Diseases, Graduate School of Medicine, The University of Tokyo, Tokyo, Japan; 11 Department of Metabolism and Nutrition, Mizonokuchi Hospital, Teikyo University, Kawasaki, Kanagawa Japan; 12 Department of Pediatrics, Graduate School of Medicine, The University of Tokyo, Tokyo, Japan; 13 Laboratory of Gene Regulation, Faculty of Medicine, University of Tsukuba, Ibaraki, Japan; 14 Biotechnology Research Institute for Drug Discovery, National Institute of Advanced Industrial Science and Technology, Tsukuba, Ibaraki, Japan; 15 TOKIWA-Bio Inc., Tsukuba, Ibaraki, Japan

**Keywords:** Human-induced pluripotent stem cells, Sendai virus vector, SeVdp-302L, Feeder-free, CD34^+^ hematopoietic stem and progenitor cells, Peripheral blood, Disease modeling, Biobank

## Abstract

**Background:**

Disease modeling with patient-derived induced pluripotent stem cells (iPSCs) is a powerful tool for elucidating the mechanisms underlying disease pathogenesis and developing safe and effective treatments. Patient peripheral blood (PB) cells are used for iPSC generation in many cases since they can be collected with minimum invasiveness. To derive iPSCs that lack immunoreceptor gene rearrangements, hematopoietic stem and progenitor cells (HSPCs) are often targeted as the reprogramming source. However, the current protocols generally require HSPC mobilization and/or ex vivo expansion owing to their sparsity at the steady state and low reprogramming efficiencies, making the overall procedure costly, laborious, and time-consuming.

**Methods:**

We have established a highly efficient method for generating iPSCs from non-mobilized PB-derived CD34^+^ HSPCs. The source PB mononuclear cells were obtained from 1 healthy donor and 15 patients and were kept frozen until the scheduled iPSC generation. CD34^+^ HSPC enrichment was done using immunomagnetic beads, with no ex vivo expansion culture. To reprogram the CD34^+^-rich cells to pluripotency, the Sendai virus vector SeVdp-302L was used to transfer four transcription factors: *KLF4*, *OCT4*, *SOX2*, and *c-MYC*. In this iPSC generation series, the reprogramming efficiencies, success rates of iPSC line establishment, and progression time were recorded. After generating the iPSC frozen stocks, the cell recovery and their residual transgenes, karyotypes, T cell receptor gene rearrangement, pluripotency markers, and differentiation capability were examined.

**Results:**

We succeeded in establishing 223 iPSC lines with high reprogramming efficiencies from 15 patients with 8 different disease types. Our method allowed the rapid appearance of primary colonies (~ 8 days), all of which were expandable under feeder-free conditions, enabling robust establishment steps with less workload. After thawing, the established iPSC lines were verified to be pluripotency marker-positive and of non-T cell origin. A majority of the iPSC lines were confirmed to be transgene-free, with normal karyotypes. Their trilineage differentiation capability was also verified in a defined in vitro assay.

**Conclusion:**

This robust and highly efficient method enables the rapid and cost-effective establishment of transgene-free iPSC lines from a small volume of PB, thus facilitating the biobanking of patient-derived iPSCs and their use for the modeling of various diseases.

**Electronic supplementary material:**

The online version of this article (10.1186/s13287-019-1273-2) contains supplementary material, which is available to authorized users.

## Background

In the past decade, thousands of human-induced pluripotent stem cells (hiPSCs) have been generated from healthy donors and from patients afflicted with various diseases [1–3]. The patient-derived hiPSCs have the potential to infinitely produce specialized disease-associated cells and organoids, enabling researchers to recapitulate some pathological aspects in Petri dishes for human disease modeling. In fact, such models have already contributed to the uncovering of molecular mechanisms of pathogenesis, potentially leading to the development of new treatments for some diseases [3]. Therefore, a simple and efficient method to establish patient-derived hiPSC lines in laboratories and biobanks is essential to facilitate the further understanding of disease pathogenesis and the development of safe and effective treatments.

Although many different methods for generating hiPSCs have been developed, they all have huge variations in their characteristics, including efficiency, quality, speed, cost, and robustness. Several non-integrating methods are currently available, using episomal DNAs [4, 5], Sendai virus (SeV) [6], adenovirus [7], PiggyBac transposons [8], minicircles [9], synthesized RNAs [10], and recombinant proteins [11] to transfer reprogramming factors into target cells. Moreover, many additional enhancers for somatic cell reprogramming have been identified and used with or without these technologies [12]. However, these technologies generally remain inefficient overall, with their reprogramming efficiencies being less than 1% in many cases [13–15]. An unstable and/or insufficient transgene expression may be a contributory factor. Prolonged residual exogenous factors may also negatively affect the high-quality generation of transgene-free hiPSCs [4, 6]. Recently, we reported the use of a novel SeV vector, SeVdp-302L, which delivers a single-stranded RNA genome containing multiple transgenes into somatic cells in a manner suitable for hiPSC generation. The SeVdp-302L vector carrying four Yamanaka factors, named SeVdp(KOSM)-302L, induces stable transgene expression and also possesses an auto-erasable feature by responding to the presence of stem cell-specific microRNA-302. Using this vector, we demonstrated the simple and efficient generation of transgene-free hiPSCs without additional reprogramming enhancers [16].

Patient-derived hiPSCs are generated from various somatic cells, the two major sources of which are skin fibroblasts [1] and peripheral blood (PB) [17]. However, skin fibroblasts require some invasive procedures for collection and have a potential risk of accumulated mutations resulting from their exposure to external stressors, such as UV rays. Indeed, undeniably high frequencies of preexisting coding mutations have been identified in both the original fibroblasts and their derived hiPSCs [18]. On the other hand, PB is thought to be a more attractive somatic cell source, since its collection is less invasive and its mutational load is presumably lower than that of skin tissue. PB is also considered useful for hiPSC generation because the cells remain viable enough at room temperature for ~ 48 h after collection [19] and after cryopreservation, enabling long-distance transport of the patient samples and scheduled hiPSC generation. For PB cell reprogramming, either lymphocytes (mostly T cells) or non-lymphocytes (e.g., stem and progenitor cells and myeloid cells) are used to generate hiPSCs. The choice depends on the aim of the studies, where lymphocytes are preferred when the specific immunoreceptor gene rearrangement is required, whereas non-lymphocytes are used for broader applications. Pioneering studies tested PB-derived CD34^+^ hematopoietic stem and progenitor cells (HSPCs) as a source for generating non-lymphocyte-derived hiPSCs, resulting in the successful establishment of such cells but with very low reprogramming efficiencies [20, 21]. Other studies used mobilized PB-derived CD34^+^ cells to obtain a sufficient number of target cells [17, 22–25], but the mobilization procedure was not suited to ill-conditioned patients in general. For the use of non-mobilized PB cells, most studies have conducted ex vivo expansion culture of the CD34^+^ HSPCs either in a way favoring general myeloid cell proliferation or erythroid cell growth, mainly to overcome their scarcity at the steady state or to enhance their overall efficiencies [19, 20, 26–37]. These cultures need a lot of cytokines, making the procedures costly and labor-intensive. Therefore, it is desirable to develop an hiPSC generation protocol that allows the use of non-mobilized PB-derived CD34^+^ cells without the need for extensive culturing, while achieving high efficiency and robustness.

Previously, we had succeeded in generating disease-specific hiPSCs from patient bone marrow-derived CD34^+^ cells or from freshly obtained PB-derived CD34^+^ cells using the SeVdp vector (the one-generation-old version of SeVdp-302L) with on-feeder conditions [38–40]. However, for the purpose of biobanking, we sought to establish a more robust method to fulfill the abovementioned requirements and to allow a feeder-free protocol for simplicity. Consequently, in this present paper, we report the successful establishment of a series of hiPSC lines from 15 patients with 8 different types of diseases, using our new protocol. This simple SeV method allows the robust and rapid establishment of transgene-free hiPSC lines from non-mobilized blood samples, with high reprogramming efficiencies (up to > 5%). The scheduled hiPSC generation was feasible because we could start a CD34^+^ cell-enrichment step from frozen mononuclear cell stocks without hampering the overall establishment procedures. No extensive expansion culture was necessary. Characterization of the hiPSC lines verified their utility for downstream applications, with their stable pluripotency and intact trilineage differentiation capability. Taken together, the results of this study indicate that this robust and highly efficient method promises the facile establishment of disease-specific hiPSC lines that are compatible with the purpose for biobanking, which will be useful for medical research.

## Methods

### Preparation of healthy donor and patient PBMC samples

A frozen vial of PB mononuclear cells (PBMCs) derived from a healthy donor was purchased from Lonza Walkersville. The other PB samples described in this report were obtained from 15 patients with 8 different diseases. Informed consent was obtained in an appropriate manner for all the cases according to the Declaration of Helsinki standard ethics procedure, with approval from the Institutional Ethical Committees (see “Ethics approval and consent to participate” section). The non-mobilized fresh PB samples from patients were collected into heparin tubes by venipuncture. Except for 2 cases, all patient PBMCs were purified with Lymphocyte Separation Medium (PromoCell) in 50-mL Leucosep™ Tubes (Greiner Bio-One) within 5 h after PB collection and were then cryopreserved in Bambanker™ freezing medium (NIPPON Genetics) for up to ~ 1 year in liquid nitrogen until use for hiPSC generation (Table 1). In the cases of patients for TkSCR1 and TkS42-1, who resided far from Tokyo, the purification and cryopreservation of their PBMCs were performed on the next day after long-distance transport of the samples to our laboratory.

**Table 1:** Information on the healthy donor and patient samples

Healthy donor and patients (hiPSC line)	Age (year)/gender	No. of cells or blood volume donated	No. of purified PBMCs per 1 mL blood sample
Healthy (TkPP2)	33/M	1.0 × 10^7^ cells	N.A.
SLE (TkSLE3)	59/F	22 mL	0.5 × 10^6^ cells/mL
SLE (TkSLE4)	50/F	30 mL	0.5 × 10^6^ cells/mL
SLE (TkSLE5)	43/F	30 mL	1.3 × 10^6^ cells/mL
PM (TkSPD3)	65/M	30 mL	0.7 × 10^6^ cells/mL
PM (TkSPD4)	45/F	30 mL	2.4 × 10^6^ cells/mL
PM (TkSPD5)	56/M	30 mL	1.1 × 10^6^ cells/mL
X-CGD (TkSCG3)	18/M	38 mL	0.8 × 10^6^ cells/mL
X-CGD (TkSCG4)	9/F	20 mL	1.2 × 10^6^ cells/mL
PID (TkSPR1)	33/F	30 mL	1.9 × 10^6^ cells/mL
PID (TkSPR2)	30/F	22 mL	1.3 × 10^6^ cells/mL
JIA (TkSCR1)	3/F	23 mL	2.4 × 10^6^ cells/mL
CMS (TkS42–1)	11/F	26 mL	0.7 × 10^6^ cells/mL
MD (TkSmD1)	59/M	40 mL	0.5 × 10^6^ cells/mL
MD (TkSmD3)	32/M	30 mL	1.1 × 10^6^ cells/mL
KCS2 (TkSKCII2)	13/F	22 mL	1.0 × 10^6^ cells/mL

The disease/symptom affecting each individual and the name of the derived human induced pluripotent stem cell (hiPSC) line are listed. The age and gender of each donor are also indicated. The number (No.) of peripheral blood mononuclear cells (PBMCs) and total peripheral blood (PB) volume donated are shown for the purchased healthy donor PBMCs and the patient PBMCs, respectively. The right-hand column shows the number of PBMCs purified from 1 mL of patient PB

*SLE* systemic lupus erythematosus, *PM* polymyositis, *X-CGD* X-linked chronic granulomatous disease, *PID* primary immunodeficiency, *JIA* juvenile idiopathic arthritis, *CMS* congenital malformation syndrome, *MD* mitochondrial diabetes, *KCS2* Kenny-Caffey syndrome type 2, *N.A* not applicable

### Preparation of CD34^+^-enriched cell population

**Fig. 1:**
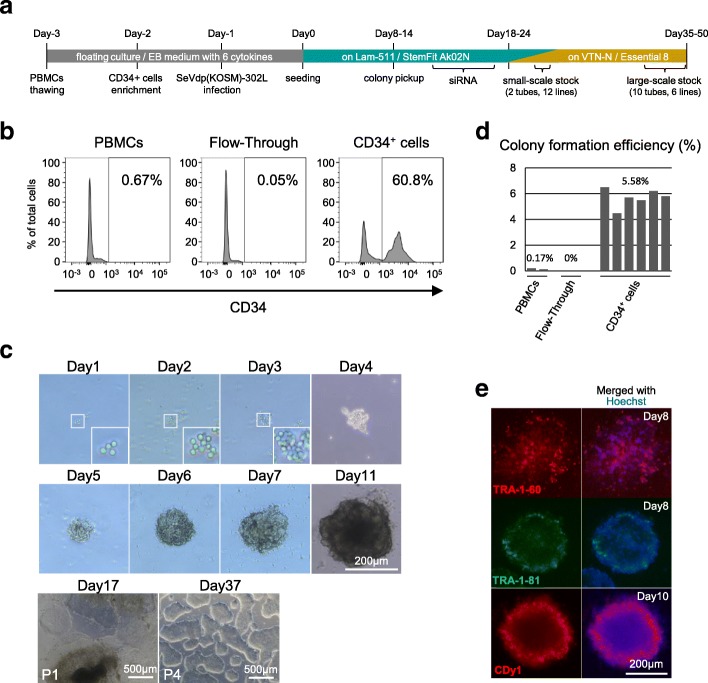
Healthy donor-derived human-induced pluripotent stem cell (hiPSC) generation from non-mobilized peripheral blood (PB)-derived CD34^+^ hematopoietic stem and progenitor cells (HSPCs) using SeVdp(KOSM)-302L. **a** Schematic diagram illustrating the schedule of hiPSC generation. **b** Percentages of cells expressing CD34, as assessed by FACS analysis of non-enriched peripheral blood mononuclear cells (PBMCs), a flow-through population (flow-through), and the CD34^+^-selected cells (CD34^+^ cells). The results demonstrate significant enrichment of the CD34^+^ cells (up to 60.8%) after immunomagnetic bead selection. **c** Sequential images of a representative colony derived from SeVdp(KOSM)-302L-transduced CD34^+^ cells, showing a phase of initial proliferation (day 1–day 4), followed by the formation of spherical colony-like structures (day 5–day 11). Also shown are images of typical hiPSC-like colonies that appeared during the subsequent expansion phase (day 17 and day 37). Magnified images are shown in insets for clarity. P1 and P4 indicate passage 1 and passage 4, respectively. **d** Colony formation efficiency of each seeded cell type. PBMCs, flow-through cells, and CD34^+^ cells were tested after infection with SeVdp(KOSM)-302L. Each bar represents the efficiency assessed in each individual well. The mean efficiency values for PBMCs (0.17%), flow-through (0%), and CD34^+^ PBMCs (5.58%) are shown. **e** Fluorescent images of live colonies stained with anti-TRA-1-60 antibody (red, top), anti-TRA-1-81 antibody (green, middle), or the CDy1 dye (red, bottom). Nuclei were stained with Hoechst 33342 dye (blue)

At day − 3, 0.4 × 10^7^ to 1.0 × 10^7^ PBMCs were thawed with ThawSTAR™ (BioCision) and kept overnight in Embryoid Body (EB) medium in 6-well plates at 37 °C with 5% CO_2_ (Fig. 1a and Table 2). The EB medium consisted of Iscove’s modified Dulbecco’s medium (Sigma) supplemented with 15% fetal bovine serum (Nichirei Biosciences), ITS liquid media supplement (Sigma), penicillin-streptomycin-glutamine (Gibco), 50 μg/mL l-ascorbic acid (Sigma), 0.45 mM 1-thioglycerol (Sigma), and the following six cytokines: 50 ng/mL stem cell factor, 50 ng/mL Fms-related tyrosine kinase 3 ligand, 10 ng/mL interleukin-3, 10 ng/mL interleukin-6, 50 ng/mL thrombopoietin, and 20 ng/mL granulocyte colony-stimulating factor (G-CSF) (all from R&D Systems). At day − 2, enrichment of the CD34^+^ cells was performed using the CD34 MicroBead Kit (Miltenyi Biotec) according to the manufacturer’s instructions. The CD34^+^-enriched PBMCs were kept overnight in EB medium in 96-well plates at 37 °C with 5% CO_2_ to ensure the recovery of truly viable cells for the subsequent reprogramming procedures (Fig. 1a).

**Table 2:** Amounts of PB, PBMCs, and CD34^+^-enriched cells used for reprogramming

Healthy donor and patients (hiPSC line)	Amounts of PB and cryopreserved PBMCs used for hiPSC generation (% of live PBMCs after thawing)		No. of CD34^+^-enriched cells for transfection
Healthy (TkPP2)	–	1.0 × 10^7^ cells	15,000
SLE (TkSLE3)	8.8 mL	0.4 × 10^7^ cells (33%)	13,000
SLE (TkSLE4)	18.4 mL	1.0 × 10^7^ cells (50%)	16,000
SLE (TkSLE5)	7.5 mL	1.0 × 10^7^ cells (71%)	39,375
PM (TkSPD3)	7.5 mL	0.5 × 10^7^ cells (52%)	11,500
PM (TkSPD4)	4.2 mL	1.0 × 10^7^ cells (61%)	12,5000
PM (TkSPD5)	8.8 mL	1.0 × 10^7^ cells (38%)	14,000
X-CGD (TkSCG3)	13.1 mL	1.0 × 10^7^ cells (53%)	24,700
X-CGD (TkSCG4)	8.5 mL	1.0 × 10^7^ cells (56%)	9000
PID (TkSPR1)	5.3 mL	1.0 × 10^7^ cells (76%)	18,000
PID (TkSPR2)	7.7 mL	1.0 × 10^7^ cells (38%)	31,000
JIA (TkSCR1)	4.2 mL	1.0 × 10^7^ cells (65%)	33,375
CMS (TkS42–1)	13.9 mL	1.0 × 10^7^ cells (21%)	5500
MD (TkSmD1)	20.0 mL	1.0 × 10^7^ cells (12%)	63,000
MD (TkSmD3)	4.7 mL	0.5 × 10^7^ cells (56%)	55,000
KCS2 (TkSKCII2)	4.8 mL	0.5 × 10^7^ cells (68%)	42,000

The amounts of peripheral blood (PB) and peripheral blood mononuclear cells (PBMCs) used in the generation of these human induced pluripotent stem cell (hiPSC) lines are listed. The PB volume shown here was calculated from the corresponding PBMC numbers contained in each frozen vial (cryopreserved PBMCs). The number of PBMCs represents the value before cryopreservation. After thawing, a certain level of cell death occurs; % live PBMCs are thus shown in parentheses, calculated by counting the actual number of living cells recovered after thawing. The number of CD34^+^-enriched cells used for SeVdp(KOSM)-302L transduction is also indicated

*SLE* systemic lupus erythematosus, *PM* polymyositis, *X-CGD* X-linked chronic granulomatous disease, *PID* primary immunodeficiency, *JIA* juvenile idiopathic arthritis, *CMS* congenital malformation syndrome, *MD* mitochondrial diabetes, *KCS2* Kenny-Caffey syndrome type 2

### Analysis of enriched CD34^+^ cells derived from PBMCs

The purchased healthy donor PBMCs were kept overnight in EB medium after thawing, following which approximately three-quarters of the cells were subjected to CD34^+^ cell enrichment as described above. Both positively selected cells and a flow-through population were obtained. The remaining one-quarter of PBMCs was left untouched as the non-treated control sample. The three samples were stained with allophycocyanin-conjugated anti-human CD34 antibody (343510, BioLegend), Pacific Blue™ dye-conjugated anti-human CD45 antibody (304022, BioLegend), and a fluorescein isothiocyanate-conjugated anti-human lineage cocktail (CD3, CD14, CD19, CD20, and CD56) (348701, BioLegend). The samples were also stained with propidium iodide to exclude dead cells. Population data were acquired with a fluorescence-activated cell sorting (FACS) Aria II sorter (BD Biosciences) and analyzed using FlowJo X software (Tree Star). A series of serially diluted CD34^+^ cells was prepared using TkSPD4 donor’s PBMCs by combining the microbead-selected samples and the flow-through population. The immunostaining and flow cytometry analysis were performed in the same way as described above.

### hiPSC generation

At day − 1, the CD34^+^-enriched cells were infected with SeVdp(KOSM)-302L at a multiplicity of infection (MOI) of 3–7 (Fig. 1a). For the experiment shown in Additional file 5: Figure S4, the CytoTune®-iPS2.0 (ID Pharma) was used to infect CD34^+^-enriched cells at an MOI of > 1, > 3, and > 6, based on the titer described in the instruction manual (> 5.0 × 10^6^ CIU/100 μL). After addition of the viral vector, the cells were left at room temperature for 2 h and then incubated overnight at 37 °C with 5% CO_2_. At day 0, the SeVdp(KOSM)-302L-infected cells were seeded into iMatrix-511 (Nippi)-coated 6-well plates (TPP) with StemFit® AK02N medium (REPROCELL) containing 10 μM Y-27632 (Fig. 1a). At day 2, half the medium volume was replaced with fresh StemFit AK02N medium, following which the entire medium was changed on every 2 days until colony pickup. At days 8–14, individual colonies with an appropriate size of 250–500 μm in diameter were picked up into iMatrix-511-coated 24-well plates (TPP) containing StemFit AK02N medium supplemented with 10 μM Y-27632 (Fig. 1a). At the medium changes on every 2 days, small interfering RNA (siRNA) treatment against SeVdp(KOSM)-302L was performed 3 times using Lipofectamine™ RNAiMAX Transfection Reagent (Invitrogen) according to the manufacturer’s instructions (Fig. 1a). The two siRNAs (siL527 and siL1913), used at 1.6 nM each, were generated from the following single-stranded RNA oligonucleotides (Fasmac): siL527-S, 5′-GGUUCAGCAUCAAAUAUGAAG-3′; siL527-AS, 5′-UCAUAUUUGAUGCUGAACCAU-3′; siL1913-S, 5′-GGUCCAGACAUGAAUUCAAAG-3′; and siL1913-AS, 5′-UUGAAUUCAUGUCUGGACCAU-3′.

### hiPSC expansion and maintenance culture for storage

For expansion and maintenance, hiPSCs were cultured on iMatrix-511- or Vitronectin (VTN-N, Gibco)-coated 12- or 6-well plates in StemFit AK02N medium (with iMatrix-511) or Essential 8™ medium (Gibco) (with VTN-N) at 37 °C with 5% CO_2_. Daily medium change was routinely performed. When passaged, the hiPSCs were mechanically and enzymatically detached from the plates using a scraper, 0.5× TrypLE™ Select (Gibco), 0.5 mM EDTA (Invitrogen), and/or Accutase® (Sigma), and plated on fresh iMatrix-511- or VTN-N-coated plates in the respective medium described above (containing 10 μM Y-27632). Before their use in the experiments, all cells were confirmed to be mycoplasma-free, using the MycoAlert™ Mycoplasma Detection Kit (Lonza) and MycoAlert™ Assay Control Kit (Lonza). Luminescence was measured with a GloMax® Explorer system (Promega). Images of hiPSC colonies were captured with a Leica DMI3000B system and their processing was performed using the ImageJ (NIH) or GNU Image Manipulation Program 2.10 (GIMP2.10). Unless we had a specific need, we generally selected ~ 12 colonies for the subsequent expansion steps that lasted for another 4–5 weeks. The small-scale frozen stocks (2 tubes) of the 12 lines were initially generated as the backup stocks, with the cells kept in the iMatrix-511/StemFit AK02N media (Fig. 1a). Six lines were adapted to culture under the VTN-N/Essential 8 conditions and finally stored as large-scale frozen stocks (10 tubes), where ~ 2.0 × 10^6^ cells per tube were cryopreserved in Bambanker for the hiPSC banking.

### RT-PCR verification of SeVdp(KOSM)-302L removal

Total RNA was isolated from the hiPSCs using the AllPrep DNA/RNA Mini Kit (Qiagen), RNeasy® Mini Kit (Qiagen), or Monarch® Total RNA Miniprep Kit (New England Biolabs). Complementary DNA (cDNA) was synthesized using the High-Capacity cDNA Reverse Transcription Kit (Applied Biosystems). The reverse transcription-polymerase chain reaction (RT-PCR) assay for SeVdp(KOSM)-302L cDNA was performed with TaKaRa Ex Taq® Hot Start Version (TaKaRa). *GAPDH* was used as the internal control. The PCR products were resolved on 2.5% agarose gels. The primers used in this study are listed in Additional file 1: Table S1.

### In vitro differentiation of three germ layers

To analyze the capability of the hiPSCs to differentiate into three germ layers, in vitro differentiation was performed with the STEMdiff™ Trilineage Differentiation Kit (STEM CELL Technologies) according to the manufacturer’s instructions.

### Quantification of pluripotency and differentiation markers with real-time PCR

cDNA was prepared as described above for quantifying the pluripotency markers octamer-binding transcription factor 4 (*OCT4*) and *NANOG* by multiplex TaqMan real-time PCR, using the Eagle Taq Master Mix with ROX (Roche). Universal probes #34 and #69 (Roche) were used for labeling the *OCT4* and *NANOG* products, respectively. The Human *G6PD* Gene Assay (Roche) was used for quantifying the reference gene. Because the universal probe #69 failed to be incorporated into the *NANOG* transcripts of the TkPP2 lines owing to the presence of a single nucleotide polymorphism, the SYBR Green method was used with the Power SYBR Green PCR Master Mix (Applied Biosystems) for these cell lines only. The relative expression of *OCT4* and *NANOG* was evaluated with reference to their expression levels in our standard hiPSC line TkDA3-4 previously established from a healthy donor [41].

The gene expression of trilineage differentiation markers was evaluated using a real-time PCR SYBR Green method with the Power SYBR Green PCR Master Mix (Applied Biosystems) or THUNDERBIRD® SYBR qPCR Mix (Toyobo). The marker genes analyzed were paired box 6 (*PAX6*), SRY-box 1 (*SOX1*), SRY-box 17 (*SOX17*), Foxhead box A2 (*FOXA2*), T-box transcription factor T (or Brachyury) (*TBXT*), and neural cell adhesion molecule (*NCAM*). *G6PD* was used as the reference gene. The primers used in this analysis are listed in Additional file 1: Table S1. All the real-time PCR analyses were performed using a CFX96 C1000™ thermal cycler (Bio-Rad). Relative expression was calculated by the ΔΔCt method with CFX™ Manager software (Bio-Rad).

### Detection of pluripotency characteristics with immunostaining and chemical staining

Live hiPSC colonies were directly stained with the NL557-conjugated anti-human TRA-1-60 antibody (NLLC4770R, R&D Systems), the LN493-conjugated anti-human TRA-1-81 antibody (NLLC16581G, R&D Systems), or the fluorescent imaging probe CDy1 [42]. Fluorescence images were captured with a Nikon Eclipse Ti microscope system. Images were edited using ImageJ (NIH) or GNU Image Manipulation Program 2.10 (GIMP2.10).

### Karyotyping

hiPSCs were expanded in VTN-N-coated 25-cm^2^ flasks (TPP) with Essential 8 medium. Standard G-banded karyotyping was performed by the Chromosome Science Laboratory or NIHON Gene Research Laboratories.

### *TCRγ* gene rearrangement analysis

Genomic DNA was isolated using the AllPrep DNA/RNA Mini Kit. Then, the rearrangement of the T cell receptor gamma (*TCRγ*) gene segment was analyzed using the T Cell Receptor Gamma Gene Rearrangement Assay Kit for Gel Detection (Invivoscribe) according to the manufacturer’s instructions.

### Flow cytometry analysis

Detailed information is provided in Additional file 2: Supplemental Methods.

## Results

### Establishing hiPSC lines from healthy donor PB-derived CD34^+^ HSPCs

We sought to establish an hiPSC generation method capable of meeting the following requirements: (1) use of non-mobilized PB cells, (2) compatibility with scheduled procedures, (3) integration-free culture, (4) absence of immunoreceptor gene rearrangement, (5) high efficiency, (6) cost-effectiveness, and (7) feeder-free conditions. We finally achieved these goals with the protocol summarized in Fig. 1a.

One example of a series of hiPSC generation processes is shown in Fig. 1b–e. In this attempt, we started from frozen healthy donor PBMCs (1.0 × 10^7^ cells) that were commercially available (Table 1). After thawing at day − 3, the PBMCs were kept overnight in an incubator under cytokine-rich conditions, mainly in order to maximize the recovery of viable HSPCs rather than to aim at cell expansion. At day − 2, CD34^+^ cell enrichment was performed using immunomagnetic beads, and the level of enrichment was checked in an independent experiment. The ~ 15,000 CD34^+^ cells enriched in this attempt (Table 2) made up ~ 60% of the final selection product (Fig. 1b). These cells were again kept overnight in a 96-well plate under the cytokine-rich culture condition to obtain only truly viable cells for the subsequent steps (Fig. 1a). At day − 1, SeVdp(KSOM)-302L was added to the cells at an MOI of 3 and the plates were incubated overnight. After these 3-day procedures, the transduced cells were seeded at various cell densities (i.e., 500, 1000, 2000, and 4000 cells/well) onto iMatrix-511-coated 6-well plates containing StemFit AK02N medium. No more hematopoietic cytokines were needed beyond day 0. After seeding, some adherent cells showed initial proliferation on days 1–3 and started forming colony-like structures on days 4–6 (Fig. 1c). Once formed, the growth of these spherical brown colonies was rapid, with their size reaching > 200 μm at day 11 (Fig. 1c). On the basis of the number of colonies counted at day 7, the colony formation efficiency was calculated to be 5.58% per cell input (Fig. 1d and Table 3), which was extremely high compared with that of previous reports (< 1% in many cases) [43, 44]. These primary colonies were confirmed to be TRA-1-60-positive and TRA-1-81-positive (Fig. 1e). They also stained positively with CDy1 dye (Fig. 1e), demonstrating that their characteristics were compatible with those of pluripotent colonies. When the non-enriched PBMCs and flow-through samples (both containing only a few CD34^+^ cells (Fig. 1b)) were used under the same conditions, colony formation barely occurred (Fig. 1d), suggesting that the CD34^+^ cells represented a preferable target for highly efficient hiPSC generation in the SeVdp(KOSM)-302L reprogramming system. The idea that the purified CD34^+^ cells are well-suited to hiPSC establishment was later confirmed by the additional experiment, the results of which showed a strong positive correlation between the proportion of CD34^+^ cells and the colony formation efficiencies (Additional file 3: Figure S1).

**Table 3:** Colony formation efficiencies and number of established lines

Healthy donor and patients (hiPSC line)	Colony formation efficiencies (No. of colonies/No. of seeded cells)	No. of colonies per 1 mL blood	No. of lines established	Recovery success rate from frozen stock (No. of lines checked)
Healthy (TkPP2)	5.58% (781/14,000)	–	10	100% (2)
SLE (TkSLE3)	2.61% (339/13,000)	38.5	12	100% (6)
SLE (TkSLE4)	0.08% (12/16,000)	0.65	4	100% (4)
SLE (TkSLE5)	3.26% (1283/39,375)	171.1	12	100% (6)
PM (TkSPD3)	1.67% (130/7750)	17.4	12	100% (5)
PM (TkSPD4)	3.17% (246/7750)	59.1	14	100% (3)
PM (TkSPD5)	0.10% (16/14,000)	1.8	12	100% (6)
X-CGD (TkSCG3)	0.29% (23/7750)	1.7	12	100% (6)
X-CGD (TkSCG4)	0.26% (23/9000)	2.7	12	100% (6)
PID (TkSPR1)	2.43% (438/18,000)	83.2	12	100% (5)
PID (TkSPR2)	2.50% (294/11,750)	38.1	12	100% (6)
JIA (TkSCR1)	1.59% (530/33,375)	126.7	11	100% (6)
CMS (TkS42–1)	0.34% (17/5000)	1.2	14	100% (6)
MD (TkSmD1)	1.09% (85/7800)	4.6	26	100% (7)
MD (TkSmD3)	1.09% (215/19,763)	45.9	36	100% (13)
KCS2 (TkSKCII2)	0.15% (43/27,750)	8.9	12	100% (2)

The colony formation efficiency of each seeded cell line and the estimated number of colonies per 1 mL of peripheral blood are shown. The ratios of the total number of emerged primary colonies/the total number of seeded cells are indicated in parentheses. The number of human induced pluripotent stem cell (hiPSC) lines established from each sample is also shown. The right-hand column shows the success rate of recovering lines from frozen stock, meaning that there were no failures in the freeze–thaw process for all the established stocks tested

*SLE* Systemic lupus erythematosus, *PM* Polymyositis, *X-CGD* X-linked chronic granulomatous disease, *PID* Primary immunodeficiency, *JIA* Juvenile idiopathic arthritis, *CMS* Congenital malformation syndrome, *MD* Mitochondrial diabetes, *KCS2* Kenny-Caffey syndrome type 2

**Fig. 2:**
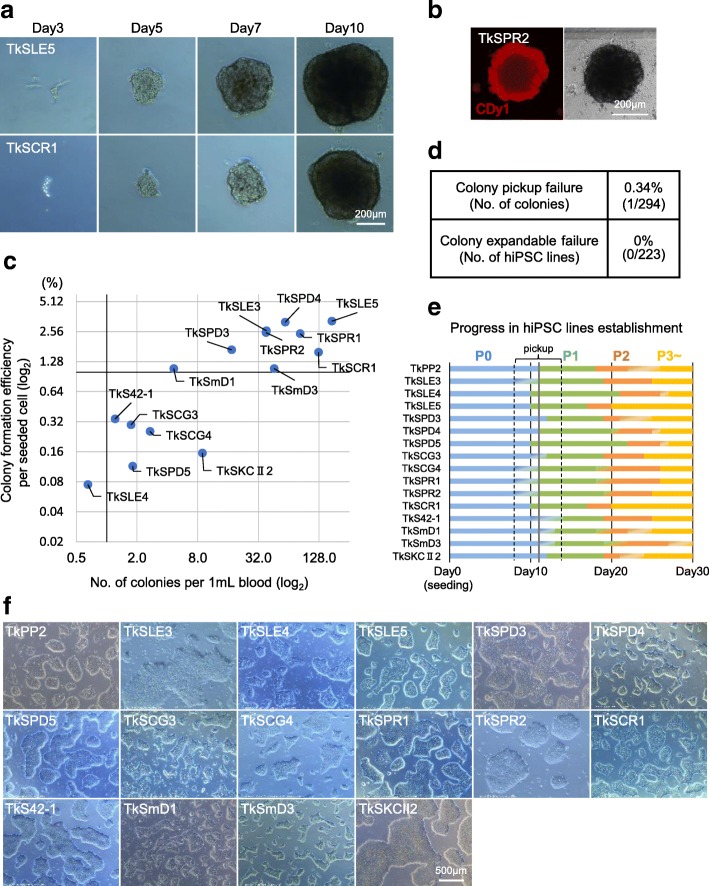
Establishment of a panel of patient-derived human induced pluripotent stem cell (hiPSC) lines from non-mobilized peripheral blood-derived CD34^+^ hematopoietic stem and progenitor cells (HSPCs) using SeVdp(KOSM)-302L. **a** Sequential images of representative primary colony growth observed in the process of TkSLE5 (top) and TkSCR1 cell line establishment (bottom). **b** Live cell image of a CDy1 dye-stained (red) representative primary colony obtained at day 13 in the course of TkSPR2 cell line generation. **c** Scatter plots indicating the colony formation efficiency of the seeded cells (y-axis) and the estimated number of hiPSC colonies obtained from 1 mL of blood (*x*-axis) from 15 patients. **d** Table showing the failure rates in the colony pickup step (top) and in the subsequent hiPSC expansion process (bottom). **e** Bar chart showing the step-by-step progress in establishing hiPSC lines from the healthy donor (TkPP2) and 15 patients. For clarity, bars are uniquely colored for each passage number: P0 (passage 0) in blue, P1 (passage 1) in green, P2 (passage 2) in orange, and P3 (passage 3) in yellow. The gray line shows a typical time point (day 11) for colony pickup, but the actual pickup dates varied between day 8 and day 14. **f** Images of the healthy donor (TkPP2)- and 15 patient-derived hiPSC lines cultured under feeder-free conditions after establishment, showing a typical monolayered colony appearance

To establish stable hiPSC lines, we tentatively performed a random pickup of 10 colonies larger than the 250-μm diameter on day 11. Their spherical shape under feeder-free conditions allowed for an extremely facile colony pickup procedure. We carried out siRNA treatment against SeVdp-302L during the initial post-pickup culture, with no colonies lost in the subsequent expansion phase. We divided each line into two feeder-free conditions (i.e., iMatrix-511/StemFit AK02N and VTN-N/Essential 8) and eventually established all 10 lines as stable hiPSCs named TkPP2 (Fig. 1c and Table 3). The colonies of the TkPP2 lines showed a typical hiPSC monolayer morphology (day 37, Figs. 1c and 2f) and were stable in culture after their recovery from cryopreservation, with their pluripotency characteristics maintained (discussed below). This trial demonstrated the high success rate of hiPSC generation from non-mobilized PB samples with no need for extensive expansion culture, allowing a rapid (5–6 weeks) and robust protocol that is appropriate for the purpose of biobanking.

### Preparation of non-mobilized PB-derived CD34^+^ HSPCs from patients

We next applied the established method to the generation of patient-derived hiPSC lines. PB samples were obtained from 15 patients of various ages (range 3–65 years) and with different types of diseases (Table 1). Among these 15 patients, there were 3 cases of systemic lupus erythematosus (SLE), 3 cases of polymyositis (PM), 2 cases of X-linked chronic granulomatous disease (X-CGD), 2 cases of primary immunodeficiency (PID), 1 case of juvenile idiopathic arthritis (JIA), 1 case of congenital malformation syndrome (CMS), 2 cases of mitochondrial diabetes (MD), and 1 case of Kenny-Caffey syndrome type 2 (KCS2) (Table 1). The detailed information of their diseases and causative genetic lesions were not provided for this report since they will be reported in more detail by collaborators elsewhere.

Using a gradient separation method, the PBMCs were obtained within a day of collection of 20–40 mL of fresh PB samples from the patients. In the JIA and CMS cases, PBMC purification was performed 1 day after PB collection owing to the need for long-distance transport of the samples. The number of PBMCs obtained ranged between ~ 1.0 × 10^7^ and ~ 7.0 × 10^7^ cells (0.45 × 10^6^ to 2.4 × 10^6^ cells/mL). These were all cryopreserved in aliquots until the start of hiPSC generation (Table 1). At day − 3, a vial containing 0.4 × 10^7^, 0.5 × 10^7^, or 1.0 × 10^7^ PBMCs was thawed for each hiPSC generation attempt, upon which it was shown that ~ 24–88% of PBMCs had been lost during the storage (Table 2). To ensure the survival of CD34^+^ HSPCs after thawing, the cells were kept overnight in EB medium containing six cytokines. At day − 2, CD34^+^ cell enrichment was performed as described for TkPP2 (Fig. 1a). The number of recovered cells after enrichment varied significantly among the patient samples, ranging from 5500 to 125,000 cells (Table 2). However, we did not experience any unsuccessful cases with these limited materials (median values; 21,350 cells).

### Successful establishment of patient-derived hiPSC lines

According to the established protocol, we infected the patient-derived CD34^+^-rich HSPCs with SeVdp(KOSM)-302L at an MOI of 3–7 on day − 1 and plated all or a part of them onto iMatrix-511-coated 6-well plates at various cell densities (i.e., 250–20,000 cells/well). Similar to the observations made with TkPP2 establishment, the attached cells gradually formed spherically shaped brown colonies containing pluripotent cells (Fig. 2a, b). The colony formation efficiencies per seeded cells showed some variation, with the average value being 1.38 ± 1.17% (mean ± SD) (Fig. 2c and Table 3). In 9 out of the 15 cases, the efficiencies varied between 1.09% and 3.26%, which translated to yields of 4.6–127.7 colonies per 1 mL of PB (Fig. 2c and Table 3). However, for the other 6 cases, the efficiency values were between 0.08% and 0.34% per seeded cells, translating to 0.65–8.9 colonies per 1 mL of PB (Fig. 2c and Table 3). The lower efficiencies in the latter group may be attributable to the putative low titer of the SeVdp(KOSM)-302L stock used for the period corresponding to the establishment, because when tested later, it was discovered that a higher MOI (i.e., 7 instead of 3) of the same virus was needed to obtain efficiencies above 1%. Nevertheless, the hiPSC generation efficiencies obtained with our method can be considered as being markedly high, even when compared with a previous report that showed an efficient reprogramming (~ 0.02%) of non-mobilized PBMCs using SeVdp(KOSM)-302L in similar feeder-free conditions [45].

Since all colonies that were randomly picked up led to the successful establishment of TkPP2 lines as described above, we performed colony pickup for the establishment of the patient-derived hiPSC lines in a similar manner, mostly on day 11 (based simply on observations under the microscope). Consequently, out of 294 colonies picked up, all but one (0.34% pickup failure) showed initial attachment and growth, enabling subsequent expansion culture. Once subjected to culture for stable hiPSC line establishment, all 223 selected colonies proved eventually to be expandable, leading to the completion of the freezing storage (Fig. 2d). The progress of the establishment processes was similar in all hiPSC lines (Fig. 2e). Furthermore, the overall process was fast, as we were able to end the culture for each line routinely within ~ 5–6 weeks with the storage of a sufficient number of hiPSCs showing typical pluripotent colonies (Fig. 2f). So far, we have not found any cases where we failed to recover viable hiPSC colonies from their frozen stocks (Table 3). Collectively, these results demonstrate that our method enables the scheduled establishment of disease-specific hiPSC lines from patient PB samples with a high success rate.

### Confirmation of the transgene-free status and karyotypes

**Fig. 3:**
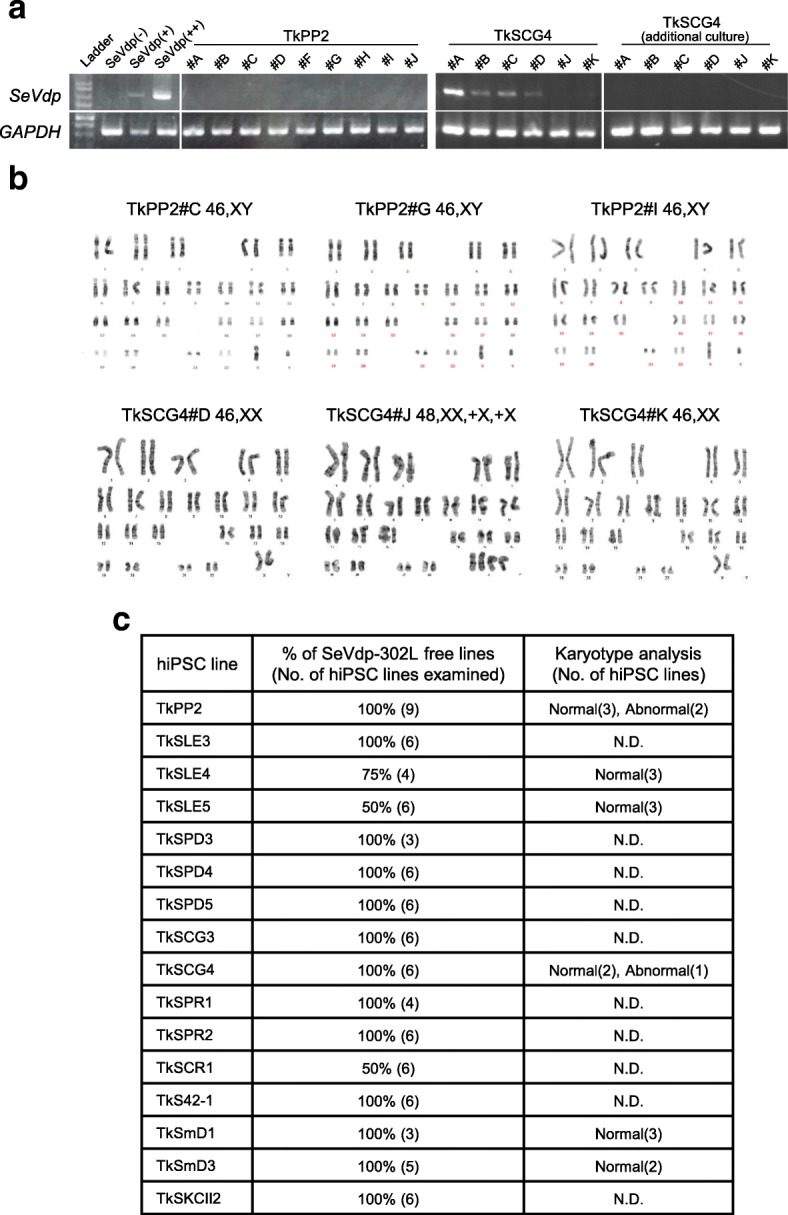
Assessment of SeVdp(KOSM)-302L removal and karyotypes. **a** Gel images showing representative assessment results for the removal of the SeVdp(KOSM)-302L genomes obtained by RT-PCR. The image demonstrates no detection of SeVdp(KOSM)-302L amplicons in all 9 TkPP2 lines, whereas it shows positive results in 4 out of 6 TkSCG4 lines (at passages 5–6). Residual SeVdp(KOSM)-302L was no longer detected in these 6 TkSCG4 lines when assessed a few passages later (additional culture). SeVdp(−), negative control (uninfected hiPSC); SeV(+), low-positive control (hiPSC carrying low copies); SeV(++), high-positive control (hiPSC carrying high copies). *GAPDH* was used as the internal control. **b** Representative G-banded karyotyping images for 3 TkPP2 lines and 3 TkSCG4 lines. **c** Summary of the assessments of SeVdp(KOSM)-302L removal and G-band karyotypes. hiPSC, human induced pluripotent stem cells; N.D., not done

To confirm the removal of SeVdp(KOSM)-302L in the established hiPSC lines, we performed RT-PCR analysis for the presence of vector genome sequences. As shown in Fig. 3a, the amplicons representing the residual existence of the SeV genome were infrequently found. This, however, occurred generally when assessed at an early passage before the initial storage. The same line showed the disappearance of detectable SeV sequences consistently with a few additional passages (Fig. 3a), most likely due to the auto-erasable function equipped in the SeVdp-302L system [16]. Most of the hiPSC lines tested were shown to be transgene-free at the time of storage (81 out of 88 lines, Fig. 3c). Karyotyping analysis has so far been conducted for a few of the patient-derived hiPSC lines and is ongoing for some others, showing mostly normal karyotypes (Fig. 3b, c). Taken together, the results indicate that our method is compatible with the measure to obtain integration-free hiPSC lines with a normal karyotype.

### Verification of a non-T lymphoid-cell origin for the hiPSCs

**Fig. 4:**
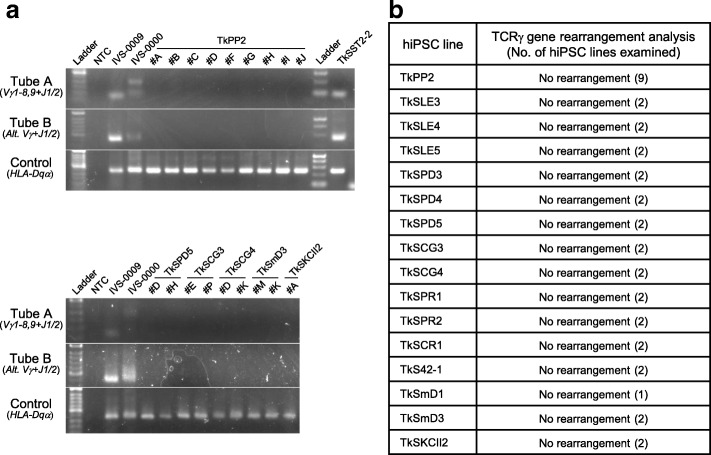
Analysis of the T cell receptor gamma (*TCRγ*) gene locus rearrangement. **a** Electrophoretic images showing the results of detection analysis for the specific *TCRγ* gene rearrangement, conducted using the T Cell Receptor Gamma Gene Rearrangement Assay kit for Gel Detection. Top: analysis of 9 independent TkPP2 lines. Bottom: analysis of 9 patient-derived human induced pluripotent stem cell (hiPSC) lines (names are indicated). NTC, non-template control. IVS-0009 and IVS-0000 are the monoclonal and polyclonal samples, respectively, provided as positive controls in the kit. Another positive control used was TkSST2-2 (top panel), a monoclonal T cell-derived hiPSC line established previously in our laboratory. Note that there are no single detectable bands in the test sample lanes. **b** Summary of the results of the *TCRγ* gene rearrangement analysis. No positive results were seen in the tested samples (38 hiPSC lines in total)

Despite the HSPC enrichment procedure, our target PBMC populations were thought to contain a significant amount of non-CD34^+^ cells, which should include lymphocytes and monocytes (Fig. 1b). hiPSCs derived from T or B cells would retain the originally rearranged immunoreceptor genome, which may be undesirable for some studies. We therefore investigated the lymphocyte-related genomic rearrangement of the *TCRγ* locus, using PCR. The *TCRγ* gene rearrangement was not detected in any of the 38 lines examined (Fig. 4a, b). The corresponding rearrangement was detectable in the T lymphoid cell-derived hiPSC line TkSST2-2, which we reported previously [46], confirming the validity of this assay (Fig. 4a). As B cells were thought to be extremely refractory to reprogramming in our system, necessitating enhancement by the coexpression of other factors (e.g., C/EBPα), an analysis of immunoglobulin genes was not conducted [47, 48]. Although the origin of monocytes cannot be excluded, these results—taken together with the great enhancement in generation efficiencies observed after CD34^+^ cell enrichment—suggest that the hiPSC lines have likely been derived from HSPCs (Fig. 1d).

### Stable pluripotency and trilineage differentiation capability

**Fig. 5:**
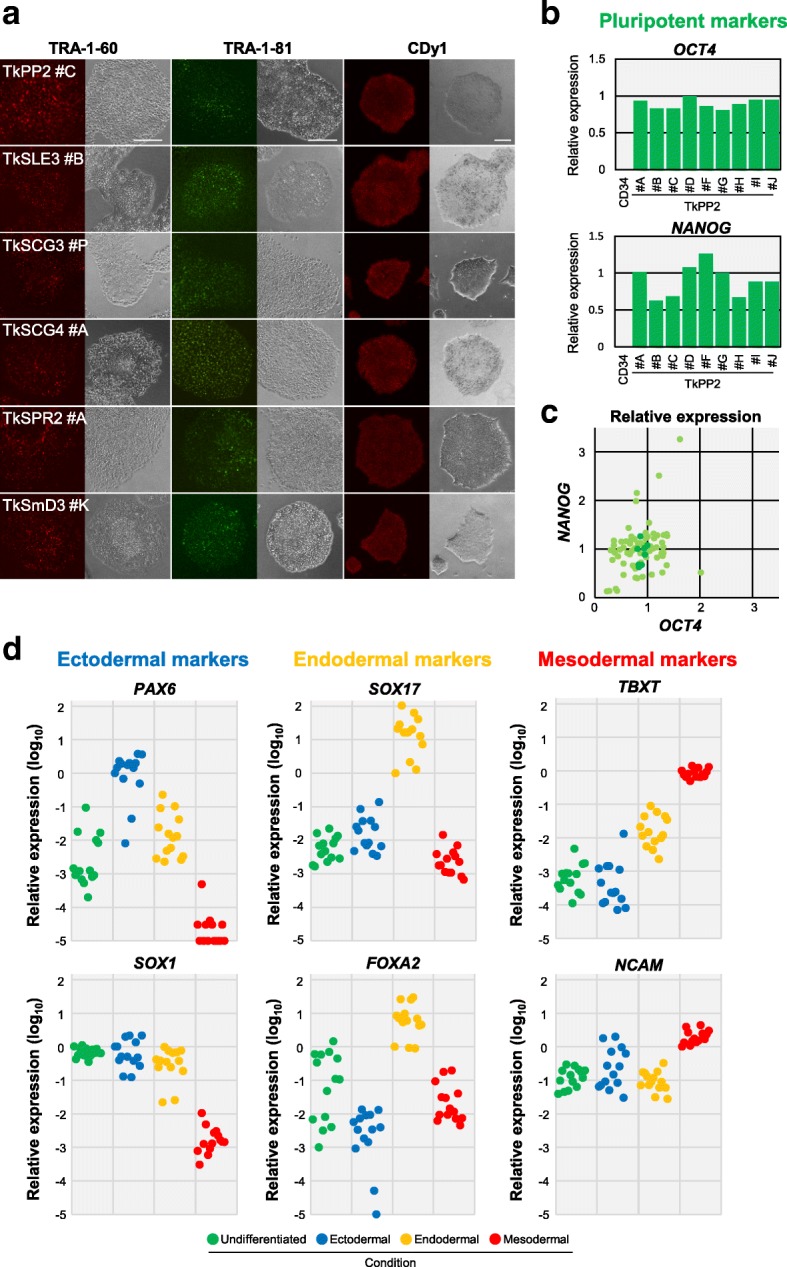
Pluripotency marker expression and trilineage differentiation capability confirmed in the established human induced pluripotent stem cell (hiPSC) lines. **a** Fluorescent images of live colonies stained with anti-TRA-1-60 antibody (red, left), anti-TRA-1-81 antibody (green, middle), or the CDy1 dye (red, right). Phase-contrast images are also shown in a side-by-side fashion for each picture. Scale bars indicate 200 μm. **b** Graph demonstrating the quantification results of *OCT4* and *NANOG* transcripts in the TkPP2 lines, assessed by RT-qPCR analysis. The relative expression values for each gene, normalized to the level observed in the standard TkDA3-4 hiPSC sample, are shown. CD34^+^-enriched cells derived from the TkPP2 donor sample were used as the negative control (CD34). **c** Scatter plot showing a comprehensive representation of the relative expression values of *OCT4* and *NANOG* in the 85 hiPSC lines examined. The results of 76 patient-derived hiPSCs are shown as light green plots. The results of 9 TkPP2 lines (same data in **b**) are shown in dark green for comparison. **d** Graphs showing the relative expression values of each marker gene calculated by the ΔΔCt method with CFX™ Manager software. The plots (14 hiPSC lines) are colored according to the culture condition shown at the bottom. “Undifferentiated” (green) represents hiPSC samples cultured in a pluripotent state. The other samples were cells cultured under differentiation conditions, with each being directed for the corresponding lineage (ectodermal, endodermal, or mesodermal)

All hiPSC lines were in a stable pluripotent state in culture, showing the retention of pluripotency characteristics, i.e., positive staining with the antibodies for TRA-1-60 or TRA-1-81 and the CDy1 dye (Fig. 5a). Expression of the marker genes *OCT4* and *NANOG* was quantified in the 85 hiPSC lines using RT-qPCR to verify their pluripotency features. As shown in Fig. 5b, c, all the lines exhibited the expression of these genes, demonstrating their pluripotent status. Expression of these markers was also confirmed at a single cell level when the samples were assessed by flow cytometry analysis (Additional file 4: Figure S2).

Finally, we examined the capability of the 14 selected hiPSC lines to differentiate into three germ layers with an easy-to-use in vitro differentiation assay. After cultivation under different conditions (i.e., either in an undifferentiated state or with differentiation for an ectodermal, an endodermal, or a mesodermal lineage), the hiPSC samples were examined by RT-qPCR for their relative gene expression of three germ-layer markers: *PAX6* and *SOX1* (ectoderm), *SOX17* and *FOXA2* (endoderm), and *TBXT* and *NCAM* (mesoderm). Selective upregulation patterns were detectable for each marker gene in accordance with the corresponding culture condition in all the hiPSC lines, except for the ectodermal marker *SOX1*, the expression levels of which were also high in the undifferentiated state (Fig. 5d). Increased expression of lineage-markers was also observed at a protein level in the differentiated TkPP2 samples by flow cytometry analysis, demonstrating the utility of this in vitro differentiation assay system (Additional file 5: Figure S3). These results confirmed that the patient-derived hiPSC lines established by our method could be stably maintained in a pluripotent culture and retain their trilineage differentiation capability, both conditions of which are mandatory for these cell lines to be truly valuable.

## Discussion

Herein, we have described a rapid and simple method for the robust establishment of transgene-free hiPSC lines using the auto-erasable SeVdp(KOSM)-302L vector [16]. In this method, we targeted non-mobilized PB-derived CD34^+^ HSPCs, with no need for cell mobilization and ex vivo expansion culture, and achieved highly efficient cell reprogramming under feeder-free conditions. Primary colonies appeared rapidly, were easily manageable for pickup, and showed robustness in the subsequent expansion steps. Using this method, we succeeded in the scheduled establishment of 223 hiPSC lines from PB samples obtained from 15 patients with 8 different diseases, generating a sufficient number of frozen stocks within 5–6 weeks. These hiPSC lines showed consistent recovery from frozen stocks, a transgene-free status, normal karyotypes, non-T cell derivation, stable pluripotency, and trilineage differentiation capability. This method is therefore valuable to robustly and efficiently generate patient hiPSC lines, allowing reductions in the cost, time, and workload for the entire procedure, which is advantageous for biobanking.

In our hiPSC banking, PB samples were used because they were easily collected from patients, with less invasiveness than that of other sources. PB can also be held at room temperature for ~ 48 h, and the derived PBMCs can be cryopreserved for a long period [19], enabling long-distance sample transport and scheduled hiPSC generation. In fact, we succeeded in generating hiPSCs from all 15 patient-derived PBMC samples that had been cryopreserved for a few months to 1 year. Of note is that despite the TkSCR1 and TkS42-1 PB samples required long-distance transport of over 24 h, they still resulted in the establishment of hiPSC lines without any detectable loss of generation efficiency (Table 3).

Among the hematopoietic cells, CD34^+^ HSPCs are often the preferred target cells for reprogramming, as they generally allow relatively highly efficient reprogramming [17, 20, 49–51]. Although CD34^+^ HSPCs can be collected from cord blood and bone marrow as well, PB is the source most suitable for patient hiPSC banking, because of its general availability. Their scarcity, however, has made it difficult to utilize PB-derived CD34^+^ cells as a regular target. To overcome this limitation, either mobilization or ex vivo expansion culture or both have been adopted for PB-derived HSPC-targeted reprogramming [22–25]. G-CSF-based mobilization, however, is considered too invasive for patient hiPSC generation. When non-mobilized PB samples are used, many protocols include cultivation steps with various combinations of cytokines to increase the target progenitor cells and thus the reprogramming efficiency [19, 21, 26–28, 30–33, 35–37, 52–54]. After expansion culture (typically 4–10 days), approximately 1.0 × 10^5^ to 1.0 × 10^7^ target cells are obtained and used for the reprogramming studies. Considering these facts, it is a marked feature that our protocol eliminates the need for both mobilization and expansion culture (Fig. 1). At present, however, we have not extensively tried to make the protocol completely cytokine-free. After immunomagnetic enrichment, the recovery of CD34^+^-rich HSPCs should be expected to be very limited because of their extremely small numbers in non-mobilized PB samples. As we continue to use frozen PBMCs for the scheduled hiPSC establishment, the expected yields would become even lower. We therefore included into the culture medium 6 cytokines that favored HSPC survival during the first 3 days. Although this treatment did not lead to a massive expansion of cells, it was nevertheless enough for successful hiPSC generation. It should be noted that our culture scale was small, thus limiting the required volume of medium (3 mL maximum on day − 3, and only 200 μL for culturing CD34^+^-enriched cells thereafter) and the total amounts of cytokines made available. To cut the cost further, we may be able to modify the cytokine-related steps in the future.

As the number of target cells was limited, our method needed reprogramming efficiencies high enough to produce a sufficient number of pluripotent colonies. To target this small number of cells (< 10,000 cells in some cases), the use of episomal vectors that have shown utility in many applications [5, 28] may not be feasible for the reason of practicality. Viral vectors are advantageous in this context; however, the current methods have revealed low reprogramming efficiencies (< 1%), even when using another type of SeV vector, and yielded some non-expandable primary colonies [19, 24, 31, 34]. In contrast, our method allowed the highly efficient generation of primary colonies (up to 5.58%) and, more importantly, enabled high success rates in the establishment of stable hiPSC lines with almost no fear of failure in the subsequent expansion steps after random pickup (Figs. 1 and 2). The hiPSC lines established showed stable pluripotency and trilineage differentiation capability (Fig. 5). We thus concluded that the special combination of SeVdp(KOSM)-302L and non-mobilized PB-derived CD34^+^ cells represented the best match, leading to the establishment of the highly successful protocol. This report has further extended the utility of SeVdp(KOSM)-302L to hiPSC generation, which has been demonstrated with other human cellular sources [45, 46, 55–59].

Besides its high efficiency, our protocol reduced the time and workload required for the entire procedure (Figs 1 and 2). Overall, a sufficient number of hiPSCs for cryopreserved stocks—which proved recoverable—were obtained within 5–6 weeks in all cases. More recently, we generally performed colony pickup at day 8 and could end the entire establishment process by day 35 after seeding in many cases. We speculated that the feeder-free condition using iMatrix-511 (Laminin-511-E8 fragment) combined with the StemFit AK02N medium might favor the rapid appearance of primary colonies and subsequent early cell growth, especially due to its great potential in supporting single-cell survival and firm cell adhesion to the plate [28, 60]. We also adapted hiPSCs to Essential 8 medium on VTN-N-coated plates in the late expansion phase for their cryopreservation (Fig. 1). This latter culture could help in the distribution of hiPSCs to other investigators, because we have found that the hiPSCs in stock are compatible with various culture conditions after thawing, irrespective of what culture systems other researchers use. The entire feeder-free protocol also benefits researchers by reducing their workload, eliminating the S23need for feeder cell handling.

Currently, researchers prefer the use of hiPSCs that are free of any genetic materials left behind after reprogramming procedures, because the residual expression of transgenes is known to affect the differentiation capability [61]. We had previously demonstrated the generation of a series of hiPSC lines using an older version of the non-auto-erasable Sendai virus vector (SeVdp), which required routine siRNA treatment to erase the SeV genome in the emerging hiPSCs [38–40]. Although we knew that SeVdp(KSOM)-302L was equipped with an auto-erasable function, we carried out siRNA treatment against SeVdp-302L to maximize the removal of the viral genomes (see the “Methods” section). Overall, the residual SeVdp-302L genome was rarely detected. Even when some residual genomes were detected, they disappeared spontaneously with a few additional cultures (Fig. 3). From these observations, we concluded that the auto-erasable function of SeVdp-302L worked efficiently in our protocol in a similar way as reported previously [16].

In this report, we described the successful establishment of an initial series of 223 hiPSC lines, derived from 15 patients affected by 8 different diseases, which included autoimmune diseases such as SLE and PD, immune deficiencies exemplified by X-CGD, and other diseases such as MD and KCS2 (Table 1). Recently, Gomez Limia et al. [62] reported the successful generation of hiPSCs from a patient, using a very similar method, where non-mobilized PB-derived CD34^+^ HSPCs with no ex vivo expansion (probably from a fresh, unfrozen sample) were reprogrammed to pluripotency under feeder-free conditions using Geltrex with the SeV vector (CytoTune® 2.0). When tested in our protocol, the CytoTune®-iPS 2.0 also yielded primary hiPSC colonies with very high efficiencies at the given setting (Additional file 6: Figure S4). Although still preliminary, the use of Sendai virus vector systems may be considered commonly compatible with our protocol. By our hands, cell line establishment is currently still ongoing, and the panel of disease-specific hiPSCs is thus expanding. Some of the hiPSC lines established in this study are already being used for disease studies by our collaborators and our research team, and the results will be reported elsewhere in the near future. For example, we have confirmed that neutrophilic cells differentiated from the TkSCG3 and TkSCG4 lines, derived from X-CGD patients, show severe defects in reactive oxygen species production (data not shown), similar to another X-CGD-iPSC line that we reported previously [39]. Such findings indicate the applicability of the established hiPSC lines to disease-modeling studies.

## Conclusions

In summary, we have developed an improved feeder-free method for establishing transgene-free hiPSC lines from non-mobilized PB-derived CD34^+^ HSPCs, using the auto-erasable SeVdp(KOSM)-302L. With this method, we successfully established a panel of disease-specific hiPSC lines from 15 patients with 8 different disease types. The hiPSC generation was feasible with the use of cryopreserved PBMCs, even after long-distance transport of the sampled PB. This method rapidly generated expandable primary colonies with high efficiency, resulting in the establishment of stable hiPSC lines with almost no failure cases. We confirmed that the hiPSC lines were suitable for use in disease modeling studies by showing their efficient transgene removal, normal karyotypes, non-T cell origin, and stable pluripotency and differentiation capability. Overall, this method allows for the scheduled generation of a panel of disease-specific hiPSC lines from patient-derived non-mobilized PB samples in a cost-effective and time- and labor-saving manner.

## Additional files

Additional file 1: **Table S1.** List of primers used in this study. (PDF 106 kb)

Additional file 2: Supplemental methods. (PDF 76 kb)

Additional file 3: **Figure S1.** Correlation between %CD34^+^ cells of the source sample and the reprogramming (colony formation) efficiency. (PDF 78 kb)

Additional file 4: **Figure S2.** Flow cytometry analysis of the marker expression in established iPSC clones. (PDF 46 kb)

Additional file 5: **Figure S3.** Flow cytometry analysis of the marker expression in the TkPP2 cells after induction of lineage-oriented differentiation. (PDF 86 kb)

Additional file 6: **Figure S4.** Comparison of CytoTune®-iPS2.0 with SeVdp(KOSM)-302L in the same hiPSC generation protocol. (PDF 87 kb)

## Data Availability

All experimental data and materials obtained and used in this study were described in this article.

## Abbreviations

BM: Bone marrow
G-CSF: Granulocyte-colony stimulating factor
hiPSC: Human-induced pluripotent stem cell
HSPC: Hematopoietic stem/progenitor cell
PB: Peripheral blood
PBMC: Peripheral blood mononuclear cell
SeV: Sendai virus
