# Supplementary material for: Robust and highly efficient hiPSC generation from patient non-mobilized peripheral blood-derived CD34+ cells using the auto-erasable Sendai virus vector
Source: Stem Cell Res Ther. 2019 Jun 24;10:185. doi: 10.1186/s13287-019-1273-2 (PMC6591940; doi:10.1186/s13287-019-1273-2)
Supplement: Supplementary file 1 — Table S1. List of primers used in this study. (PDF 106 kb) [file 13287_2019_1273_MOESM1_ESM.pdf]

Table S1. List of primers used in this study

Target name	Forward primer sequence (5’→3’)	Reverse primer sequence (5’→3’)
SeVdp	AGACCCTAAGAGGACGAAGA	ACTCCCATGGCGTAACTCCATAGTG
GAPDH	AACAGCCTCAAGATCATCAGC	TTGGCAGGTTTTTCTAGACGG
OCT4	TGCAGAAAGAACTCGAGCAA	AGATGGTCGTTTGGCTGAAT
NANOG	ATGCCTCACACGGAGACTGT	CAGGGCTGTCCTGAATAAGC
PAX6	GTCCATCTTTGCTTGGGAAA	TAGCCAGGTTGCGAAGAACT
SOX1	CACAACTCGGAGATCAGCAA	GGTACTTGTAATCCGGGTGC
BRACHYURY	AATTGGTCCAGCCTTGGAAT	CGTTGCTCACAGACCACA
NCAM	ATGGAAACTCTATTAAAGTGAACCTG	TAGACCTCATACTCAGCATTCCAGT
SOX17	CAGCAGAATCCAGACCTGCA	GTCAGCGCCTTCCACGACT
FOXA2	GGAGCGGTGAAGATGGAA	TACGTGTTCATGCCGTTCAT
