# Supplementary material for: Robust and highly efficient hiPSC generation from patient non-mobilized peripheral blood-derived CD34+ cells using the auto-erasable Sendai virus vector
Source: Stem Cell Res Ther. 2019 Jun 24;10:185. doi: 10.1186/s13287-019-1273-2 (PMC6591940; doi:10.1186/s13287-019-1273-2)
Supplement: Supplementary file 2 — Supplemental methods. (PDF 76 kb) [file 13287_2019_1273_MOESM2_ESM.pdf]

## Supplemental Methods

**Robust and highly efficient hiPSC generation from patient non-mobilized peripheral blood-derived CD34$^+$ cells using the auto-erasable Sendai virus vector**

Takashi Okumura, Yumi Horie, Chen-Yi Lai, Huan-Ting Lin, Hirofumi Shoda, Bunki Natsumoto, Keishi Fujio, Eri Kumaki, Tsubasa Okano, Shintaro Ono, Kay Tanita, Tomohiro Morio, Hirokazu Kanegane, Hisanori Hasegawa, Fumitaka Mizoguchi, Kimito Kawahata, Hitoshi Kohsaka, Hiroshi Moritake, Hiroyuki Nunoi, Hironori Waki, Shin-ichi Tamaru, Takayoshi Sasako, Toshimasa Yamauchi, Takashi Kadowaki, Hiroyuki Tanaka, Sachiko Kitanaka, Ken Nishimura, Manami Ohtaka, Mahito Nakanishi, and Makoto Otsu

### Flow cytometry analysis.

Cell surface staining was performed using the monoclonal antibodies shown below.

- APC-conjugated mouse anti-SSEA4 (BioLegend, #330418)
- PE-conjugated recombinant anti-TRA-1-60 (Miltenyi, #130-100-350)
- Alexa Fluor 488-conjugated mouse anti-NESTIN (ThermoFisher, # 53-9843-82)
- APC/Cy7-conjugated mouse anti-CXCR4 (BioLegend, #306527)
- FITC-conjugated mouse anti-NCAM (BioLegend, # 318303).

For staining of intracellular antigen, the following antibodies (monoclonal, otherwise stated) were used.

- PE-conjugated mouse anti-OCT3/4 (BD Pharmingen, #560186)
- Alexa Fluor-647-conjugated mouse anti-NANOG (BD Pharmingen, #561300)
- Alexa Fluor-647-conjugated mouse anti-PAX6 (BD Pharmingen, #562249)
- APC-conjugated goat anti-SOX17 (polyclonal, R&Dsystems, IC1924A)
- APC-conjugated goat anti-Brachyury (polyclonal, R&Dsystems, IC2085A)

To fix/permeabilize test samples, the Transcription Factor Buffer Set (BD Pharmingen, #562574) was used according to the manufacturer's instructions.

The data were acquired with a fluorescence-activated cell sorting (FACS) Aria II sorter (BD Biosciences) and analyzed using FlowJo software (Tree Star, Ashland, OR).
