# Supplementary material for: Robust and highly efficient hiPSC generation from patient non-mobilized peripheral blood-derived CD34+ cells using the auto-erasable Sendai virus vector
Source: Stem Cell Res Ther. 2019 Jun 24;10:185. doi: 10.1186/s13287-019-1273-2 (PMC6591940; doi:10.1186/s13287-019-1273-2)
Supplement: Supplementary file 3 — Figure S1. Correlation between %CD34+ cells of the source sample and the reprogramming (colony formation) efficiency. (PDF 78 kb) [file 13287_2019_1273_MOESM3_ESM.pdf]

Figure S1

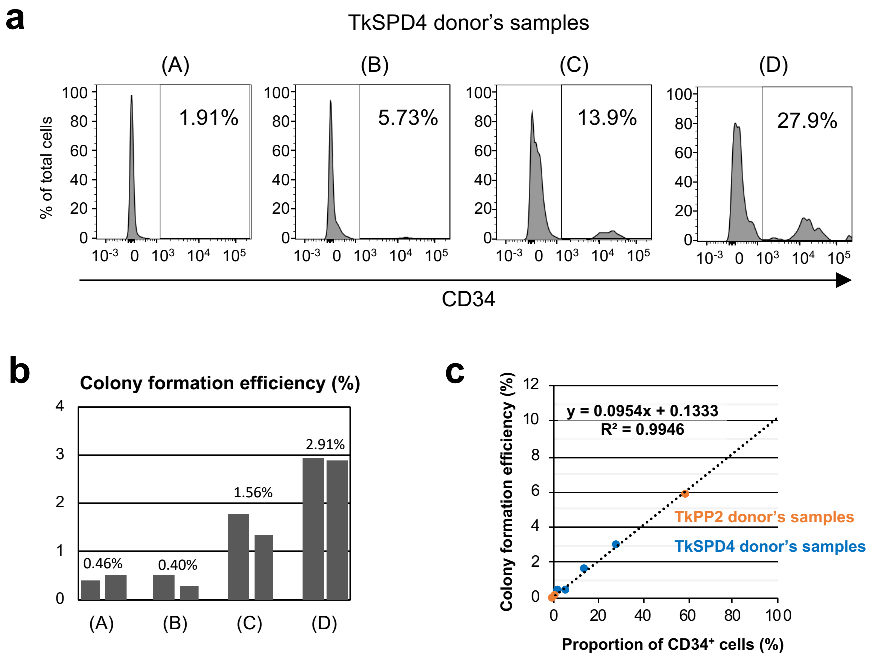

Figure S1 Correlation between %CD34⁺ cells of the source sample and the reprogramming (colony formation) efficiency.

a. Preparation of a series of serially diluted PBMC samples containing the TkSPD4 donor-derived CD34⁺ cells. The values represent the proportion of CD34-positive cells assessed by flow cytometry analysis in the four samples; (A) 1.91%, (B) 5.73%, (C) 13.9%, and (D) 27.9%.

b. Shown are the iPSC colony formation efficiencies per seeded cells obtained with the four samples prepared in **a.** infected by SeVdp(KOSM)-302L at MOI = 3. Each bar represents the efficiency assessed in individual well (tested in duplicate). The mean efficiency values are indicated.

c. Graphic representation of the correlation between a proportion of CD34⁺ cells and colony formation efficiency. Plots in blue are from results shown in **a.** and **b.**, with which alone a strong positive correlation is evident between two values ($y = 5.0583x + 4.105$, $R^2 = 0.9742$). When combined with the data obtained from TkPP2 samples shown in Fig. 1b and 1d (plots in orange), the correlation becomes even stronger as shown. Considering the strong positive correlation between two values, it can be presumed that the colony formation efficiency would reach as high as ~10% at MOI = 3 with the SeVdp(KOSM)-302L system, if a ~100% pure CD34⁺ cell population is used.
