# Supplementary material for: Robust and highly efficient hiPSC generation from patient non-mobilized peripheral blood-derived CD34+ cells using the auto-erasable Sendai virus vector
Source: Stem Cell Res Ther. 2019 Jun 24;10:185. doi: 10.1186/s13287-019-1273-2 (PMC6591940; doi:10.1186/s13287-019-1273-2)
Supplement: Supplementary file 4 — Figure S2. Flow cytometry analysis of the marker expression in established iPSC clones. (PDF 46 kb) [file 13287_2019_1273_MOESM4_ESM.pdf]

Figure S2

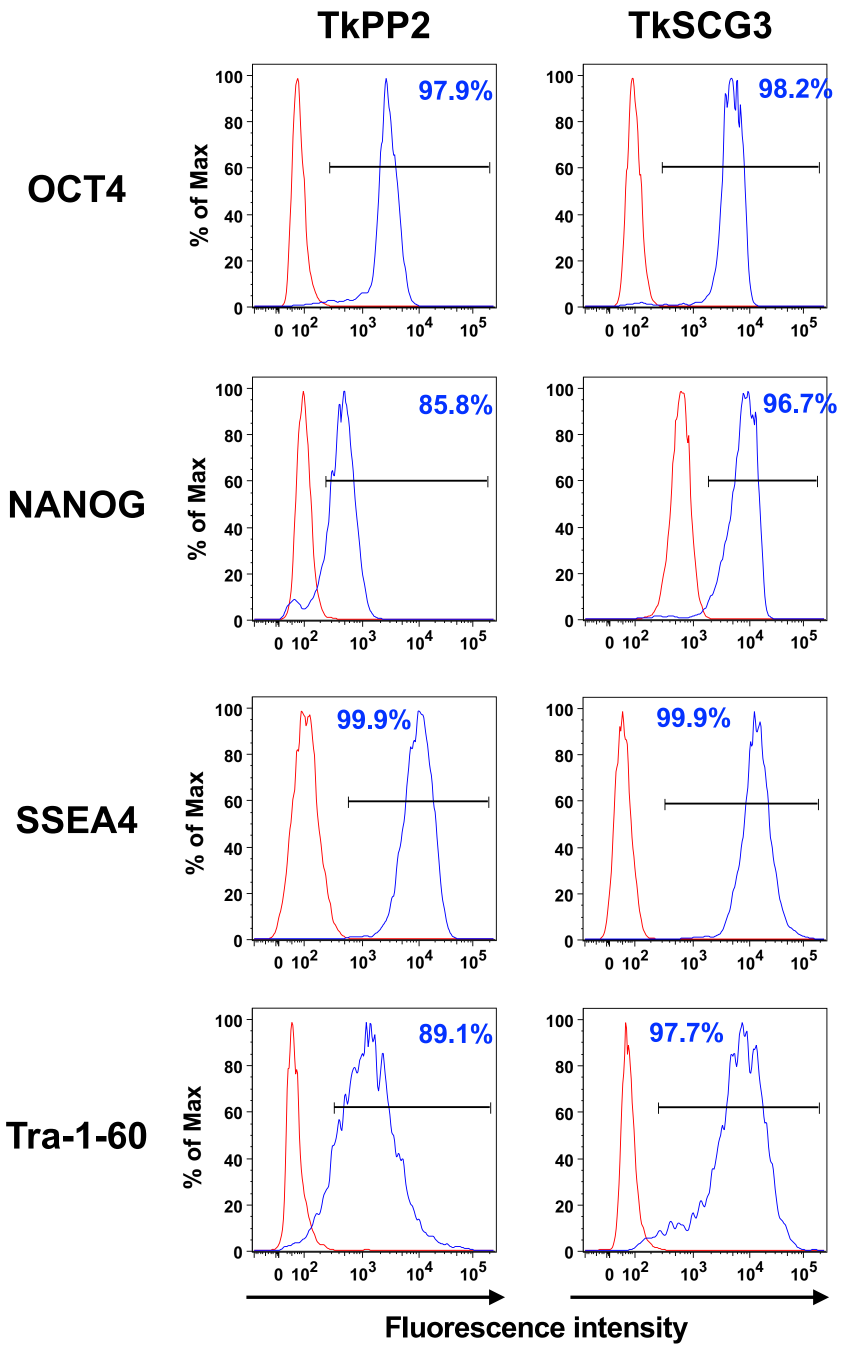

Figure S2 Flow cytometry analysis of the marker expression in established iPSC clones. Expression of each marker was assessed either intracellularly (OCT4 and NANOG) or on the cell surface (SSEA4 and Tra-1-60) in the indicated iPSC clones. Red histograms, isotype control; blue histograms, stained with the antibodies specific to each marker. Percentages are indicated in blue for the cells residing within a gate shown in black.
