# Supplementary material for: Robust and highly efficient hiPSC generation from patient non-mobilized peripheral blood-derived CD34+ cells using the auto-erasable Sendai virus vector
Source: Stem Cell Res Ther. 2019 Jun 24;10:185. doi: 10.1186/s13287-019-1273-2 (PMC6591940; doi:10.1186/s13287-019-1273-2)
Supplement: Supplementary file 5 — Figure S3. Flow cytometry analysis of the marker expression in the TkPP2 cells after induction of lineage-oriented differentiation. (PDF 86 kb) [file 13287_2019_1273_MOESM5_ESM.pdf]

Figure S3

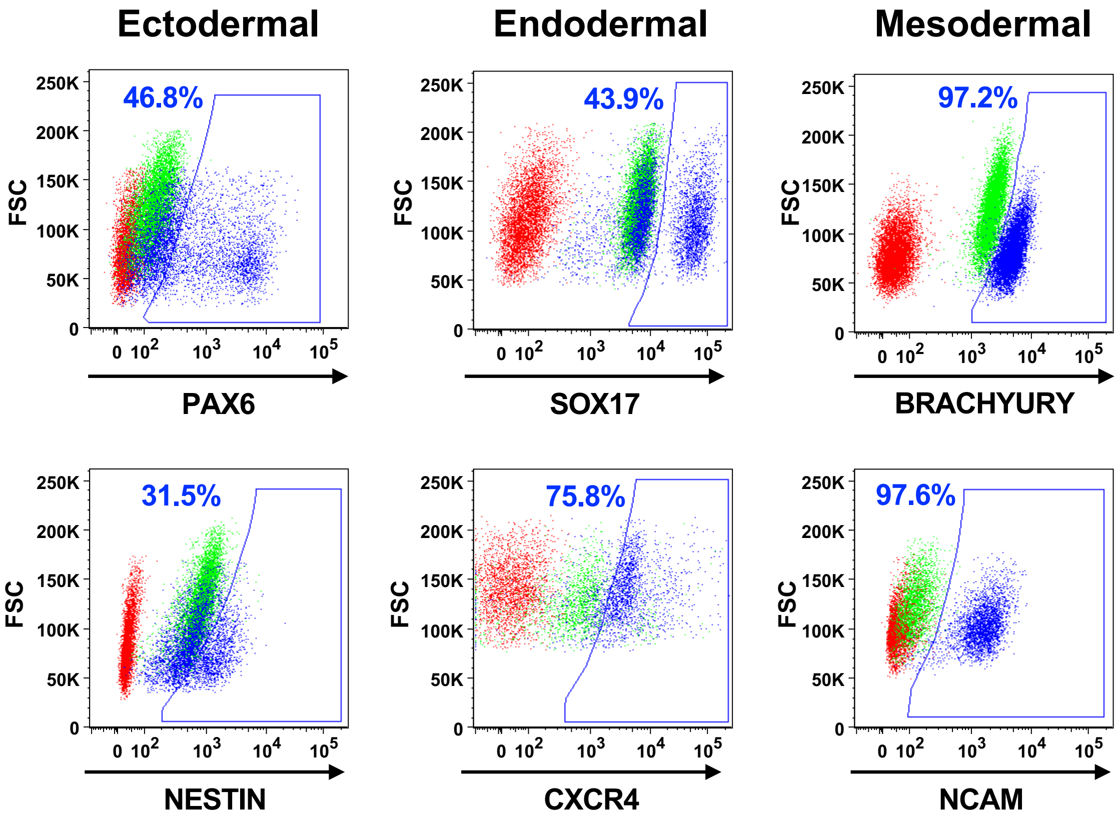

Figure S3 Flow cytometry analysis of the marker expression in the TkPP2 cells after induction of lineage-oriented differentiation.

The TkPP2 #C cells were induced to differentiate into the indicated lineage (Ectodermal, Endodermal, or Mesodermal) in culture using the ready-to-use *in vitro* differentiation kit. Six (Endodermal and Mesodermal) or 8 (Ectodermal) days later, cells were subjected to flow cytometry analysis. Top, intracellular marker expression; bottom, cell surface marker expression. Red, unstained control; blue, stained for each marker. For comparison, the plots of undifferentiated TkPP2 #C iPSCs stained for each marker are shown as control in green. Percentages are indicated in blue for the cells residing within a gate set against the undifferentiated control.
