# Supplementary material for: Robust and highly efficient hiPSC generation from patient non-mobilized peripheral blood-derived CD34+ cells using the auto-erasable Sendai virus vector
Source: Stem Cell Res Ther. 2019 Jun 24;10:185. doi: 10.1186/s13287-019-1273-2 (PMC6591940; doi:10.1186/s13287-019-1273-2)
Supplement: Supplementary file 6 — Figure S4. Comparison of CytoTune®-iPS2.0 with SeVdp(KOSM)-302L in the same hiPSC generation protocol. (PDF 87 kb) [file 13287_2019_1273_MOESM6_ESM.pdf]

Figure S4

a

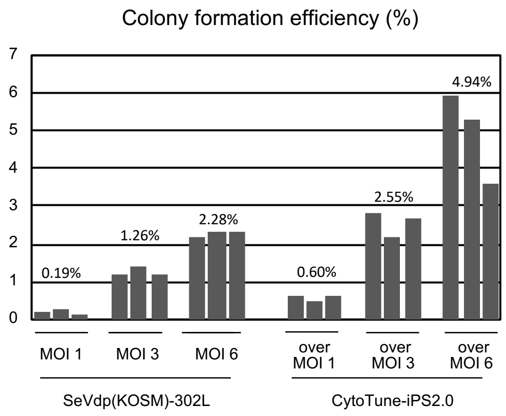

b

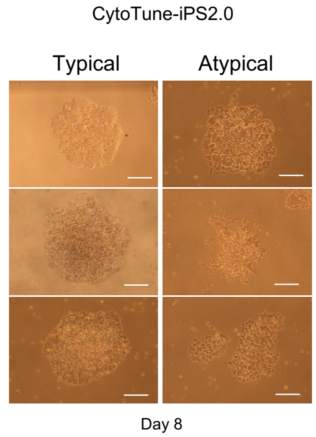

Figure S4 Comparison of CytoTune®-iPS2.0 with SeVdp(KOSM)-302L in the same hiPSC generation protocol.

a. Colony formation efficiency per seeded cells (7 days post seeding). The TkPP2 healthy donor $CD34^+$-selected cells were infected by SeVdp(KOSM)-302L and CytoTune®-iPS2.0 at the indicated MOI. Because the CytoTune® kit provides only the titer with the minimum value (*i.e.*, > 5 x 10^6 CIU / 100 µl), "over MOI" is used for description. Each bar represents the efficiency assessed in individual well. The mean efficiency values are shown (n = 3).

b. The appearance of primary colonies in CytoTune®-iPS2.0-based reprogramming. Most of the colonies showed a typical monolayer iPSC-like morphology (Typical), whereas some (~20%) emerged with an atypical appearance with certain heterogeneity (Atypical). Scale bars represent 200 µm.
